# Adiposity associates with lower plasma resolvin E1 (Rve1): a population study

**DOI:** 10.1038/s41366-024-01482-x

**Published:** 2024-02-12

**Authors:** Anne Barden, Sujata Shinde, Lawrence J. Beilin, Michael Phillips, Leon Adams, Steffen Bollmann, Trevor A. Mori

**Affiliations:** 1 Medical School, University of Western Australia, Perth, WA Australia; 2 Centre for Medical Research, University of Western Australia, Perth, WA Australia; 3 Royal Perth Hospital Research Foundation, Perth, WA Australia; 4 School of Information Technology and Electrical Engineering, Centre for Advanced Imaging, The University of Queensland, Brisbane, QLD Australia

**Keywords:** Endocrinology, Biological techniques, Risk factors

## Abstract

**Background:**

Inadequate inflammation resolution may contribute to persistent low-grade inflammation that accompanies many chronic conditions. Resolution of inflammation is an active process driven by Specialized Pro-resolving Mediators (SPM) that derive from long chain n-3 and n-6 fatty acids. This study examined plasma SPM in relation to sex differences, lifestyle and a broad range cardiovascular disease (CVD) risk factors in 978, 27-year olds from the Australian Raine Study.

**Methods:**

Plasma SPM pathway intermediates (18-HEPE, 17-HDHA and 14-HDHA), and SPM (E- and D-series resolvins, PD1, MaR1) and LTB_4_ were measured by liquid chromatography-tandem mass spectrometry (LCMSMS). Pearson correlations and multiple regression analyses assessed relationships between SPM and CVD risk factors. Unpaired t-tests or ANOVA assessed the effect of sex, smoking, unhealthy alcohol consumption and obesity on SPM.

**Results:**

Women had higher 17-HDHA (*p* = 0.01) and lower RvE1 (*p* < 0.0001) and RvD1 (*p* = 0.05) levels compared with men. In univariate analysis, obesity associated with lower RvE1 (*p* = 0.002), whereas smoking (*p* < 0.001) and higher alcohol consumption (*p* < 0.001) associated with increased RvE1. In multiple regression analysis, plasma RvE1 was negatively associated with a range of measures of adiposity including BMI, waist circumference, waist-to-height ratio, abdominal subcutaneous fat volume, and skinfold thicknesses in both men and women.

**Conclusion:**

This population study suggests that a deficiency in plasma RvE1 may occur in response to increasing adiposity. This observation could be relevant to ongoing inflammation that associates with CVD and other chronic diseases.

## Background

Low-grade chronic inflammation is a feature of chronic conditions such as the metabolic syndrome, type 2 diabetes mellitus, non-alcoholic fatty liver disease, and cardiovascular disease (CVD) [1, 2]. Evidence of a positive association between low-grade inflammation and obesity and insulin resistance suggests that inflammation is a part of a continuum of risk for diabetes and CVD [3–8]. Ongoing inflammation may in part be due to inadequate resolution that is an active process involving Specialized Pro-resolving Mediators (SPM) of inflammation resolution that act to promote a return to homeostasis [9]. SPM include lipoxins that derive from arachidonic acid (AA), and E-and D-series resolvins and maresins that derive from the n-3 fatty acids eicosapentaenoic acid (EPA) and docosahexaenoic acid (DHA), respectively [9–12]. In addition to their effects on inflammatory responses, SPM may regulate other pathways involved in the development of CVD. Studies in animal models have shown that administration of RvD1 improves insulin sensitivity and decreases adipose inflammation [13]. SPM have been implicated as important in regulating the atherosclerotic process with studies in animal models showing that administration of SPMs reduces atheroprogression (RvE1, RvD1, RvD2, Mar1), myocardial infarct size (RvD1, RvE1) and neointimal hyperplasia (RvD1, RvD2, Mar-1) [14].

In controlled studies in humans, we have shown that supplementation with n-3 fatty acids led to an increase plasma SPM pathway intermediates 18-HEPE and 17-HDHA [12, 15], that was attenuated in overweight participants with features of the metabolic syndrome [16]. Weight loss in those with the metabolic syndrome associated with a two-fold increase in RvE1 from stimulated neutrophils [17]. Together, these findings suggest that excess body fat may impair the ability to synthesize SPM.

To date studies in humans examining SPM and their pathway intermediates have been relatively small, precluding the study of associations with lifestyle and cardiovascular risk factors with any confidence. This study examined plasma levels of SPM and their pathway intermediates in relation to sex differences, fatty acid status, and a range of relevant lifestyle and cardiovascular risk factors, in 978 community dwelling young adults from the Raine Study at 27-years of age.

## Methods

The Raine Study recruited 2900 pregnant women from the general population at King Edward Memorial Hospital (Subiaco, Western Australia) and nearby private practices from 1989 to 1991 [18]. The women gave birth to 2868 live babies (Generation 2, Gen2). This study focuses on the 27-year follow-up of Gen2 (Gen2-27) in which 1084 participants volunteered, with 978 providing blood samples for measurement of SPM. The Human Research Ethics Committee of the University of Western Australia approved the study. Participation was voluntary and informed written consent was obtained.

### Clinical and laboratory measurements

Body weight (to the nearest 100 g) and height (to the nearest 0.1 cm) were measured using a Wedderburn Chair Scale and a Holtain Stadiometer, respectively. BMI was calculated as weight (kg)/height^2^ (m). Waist circumference was measured at the umbilicus level with a tape measure (to the nearest 0.1 cm). Skinfold thickness measurements were obtained from the anterior abdominal wall, subscapular, and suprailiac skinfolds with a skinfold caliper (Holtain Tanner/ Whitehouse skinfold caliper, Holtain, Crosswell, United Kingdom). Resting systolic blood pressure and diastolic blood pressure were measured after 5 min supine rest using an oscillometric sphygmomanometer (DINAMAP ProCare 100 vital signs monitor; GE Healthcare, USA). Six readings were recorded, each 2 min apart, with the last five readings averaged.

Fasting bloods were analyzed at PathWest Laboratory, Royal Perth Hospital, for serum insulin, glucose, triglycerides, total cholesterol, HDL-cholesterol, high-sensitivity C-reactive protein (hs-CRP), the liver enzymes gamma-glutamyl transferase, alanine transaminase, aspartate transaminase and alkaline phosphatase, and leukocyte count. LDL-cholesterol was calculated using the Friedewald equation [19]. Insulin resistance was estimated using HOMA-IR calculated as fasting insulin [µU/ml] × fasting glucose [mmol/L]/22.5). Fatty acids were measured in erythrocytes that were collected and stored at −80 ^o^C until assay [20]. Plasma leptin and adiponectin were measured in plasma stored at −80 ^o^C using Quantitkine ELISA kits for Human Leptin and Human Total Adiponectin/ Arcp30, respectively (R&D systems Inc, Minniapolis USA). The metabolic syndrome (MetS) was defined using the criteria of the International Diabetes Foundation [21].

#### Lifestyle measures

Self-reported measures of smoking were dichotomized as a ‘yes/no’ response. Alcohol consumption and physical activity were calculated from self-reported diaries from the previous week [22]. Alcohol consumption was further dichotomized to <100grams/week or >100grams/week based on the ‘Australia guidelines to reduce health risks from drinking alcohol’ [23]. The use of hormonal contraception in females was assessed from a questionnaire that inquired about the current use of the oral contraceptive pill, implant, injection or any intrauterine hormonal contraceptive devices.

#### Assessment of abdominal body fat using magnetic resonance imaging

Subcutaneous and visceral abdominal fat depots were measured with a Siemens Magnetom Espree 1.5 T (Siemens AG, Erlangen, Germany) (76 × 18 Channels, Max Slew Rate 170 T/m/s) magnetic resonance imager (MRI) under the supervision of Dr Brendan Adler at Envision Medical Imaging, Perth. Participants were screened to ensure they had no contraindications to MRI prior to their scan. They were studied in the supine position with radiofrequency coils placed in the area being imaged. Images were acquired using a Multiecho Gradient Echo sequence, with a breath-hold technique. Data were analyzed at the Center for Advanced Imaging, University of Queensland. The DICOM images were converted to NIfTI files to facilitate data processing. After registering and histogram-matching the upper and lower abdomen segments, the two scans were merged to one full abdomen image. The full abdomen image was segmented into subcutaneous and visceral fat compartments using the method “vatsatseg”, a python implementation of the matlab segmentation tool “SAT_VAT_segmentation” as described by Shen et al. [24].

#### Measurement of SPM

SPM pathway intermediates (18-HEPE, 17-HDHA and 14-HDHA), SPM and LTB_4_ were extracted from plasma collected into EDTA and analyzed by liquid chromatography-tandem mass spectrometry (LCMSMS) as previously described [15, 25]. Standards 14-HDHA; 18-HEPE; 17S-HDHA; RvE1; RvD1; 17R-RvD1; RvD2; 10S,17S-DiHDHA; MaR1; LXA_4_; LTB_4_; and RvD2-d5 were purchased from Cayman Chemicals (Ann Arbor, MI). PD1; RvE2; RvD3; and RvD5 standards were a gift from Professor Charles N. Serhan (Harvard Medical School, Boston, MA). RvE3 and 18R-RvE3 standards were a gift from Professor Makoto Arita (Department of Health Chemistry, Graduate School of Pharmaceutical Sciences, University of Tokyo, Japan).

SPM were analyzed on a Thermo Scientific TSQ Altis Triple Quadrupole mass analyzer equipped with a HESI source attached to a Vanquish Ultra High Performance Liquid Chromatography (UHPLC) system. UHPLC separation was carried out on a Thermo Accucore C18 column (100 mm×2.1 mm, 2.6 μm particle size) at a flow rate of 400 μl/min with 5 mM ammonium acetate, pH 8.9 (solvent A) and methanol (solvent B) as mobile phases. Buffer was freshly prepared and pH was adjusted using 25% ammonia solution. All chemicals and solvents used were LCMS grade. Gradient conditions were as follows: 50% B at 0 min to 65% B at 8.2 min, 95% B at 9.2 min and held at 95% B to 11.0 min, reduced to 50% B at 12.0 min and held at 50% B to 15 min to equilibrate to starting conditions. The retention times were: RvE1 = 0.95 min, RvD2-d5 = 2.2 min, RvD2 = 2.02 min, RvD3 = 2.17 min, RvD1 = 2.56 min, LXA4 = 2.50 min, 17R-RvD1 = 2.69 min, RvE2 = 3.45 min, RvE3 = 3.53 min, PD1 = 5.26 min, 10 S,17S-DiHDHA=4.77 min, RvD5 = 5.18 min, MaR1 = 5.28, LTB4 = 5.40 min, 18-HEPE = 6.99 min, 17-HDHA = 9.62 min, 14-HDHA = 9.68 min. The total run time was 15 min.

The mass spectrometer was operated in the negative ion multiple reaction monitoring (MRM) mode using argon as collision gas. Nitrogen was used as the sheath, auxillary and sweep gas set to 50, 10 and 1 arbitrary units, and argon as the CID gas at 1.5 mTorr. The vaporizer temperature of the ESI source was 350 °C and the spray voltage was 2.5 KV. The Q1 and Q3 mass resolution of the spectrometer was 0.7 Da at full width at half maximum. SPM pathway intermediates, SPM and LTB_4_ were identified using all of the following criteria: (i) retention time that matched the authentic standard; (ii) MRM using two or for most SPM three product ions identified from the standards and optimized for collision energy ; and (iii) confirmation of retention time and MRM product ions in stripped plasma with added SPM standard. The MRM transitions were (precursor ion→product ions): 14-HDHA (*m/z* 343.175 → 281.167, 205.083, 161.083); 17-HDHA (*m/z* 343.188 → 281.167, 245.083, 201.155); 18-HEPE (*m/z* 317.200 → 259.137, 255.167, 215.137); LXA4 (*m/z* 351.188 → 235.137, 217.137, 114.970); LTB4 (*m/z* 335.175 → 317.167, 195.054, 151.054); RvE1 (*m/z* 349.125 → 205.000, 195.054, 161.071); RvE2 (*m/z* 333.175 → 315.208, 271.167, 253.167); RvE3 (*m/z* 333.225 → 315.167, 245.083, 201.167); 18R-RvE3 (*m/z* 333.188 → 315.220, 245.155, 201.155); RvD1 (*m/z* 375.200 → 233.155, 215.083, 141.054); 17R-RvD1 (*m/z* 375.150 → 233.155, 215.083, 141.071); RvD2 (*m/z* 375.200 → 215.137, 175.071, 141.000); RvD2-*d*5 (*m/z* 380.200 → 277.083, 175.071, 141.000); RvD3 (*m/z* 375.138 → 180.857, 147.054, 137.071); RvD5 (*m/z* 359.125 → 341.167, 297.167, 199.155); 10 S,17S-DiHDHA (*m/z* 359.212 → 206.083, 153.071); PD1 (*m/z* 359.162 → 341.238, 206.083, 153.054); MaR1 (*m/z* 359.212 → 341.167, 297.167, 177.054). Full mass spectrometry spectra are provided in Supplementary Figs. 1–6. Instrument control and data acquisition used Tracefinder software, version 4.1. Plasma concentrations expressed in pg/mL were determined from calibration curves constructed by spiking stripped plasma with standards and used linear regression analysis and the ratio of metabolite to internal standard (RvD2-*d*5) (typically *R*^2^ > 0.99). The % CV for all measured metabolites ranged from 5–15%.

#### Statistical analysis

Statistical analysis was carried out using SPSS version 27.0 or STATA version 15.1. SPM pathway intermediates, SPM and other variables that were not normally distributed were natural log transformed prior to analysis. Participant characteristics are described as arithmetic means and standard deviations or geometric means and 95% confidence intervals.

Sex differences in participant characteristics were assessed using unpaired t-test or Chi square analysis for categorical variables. Correlation coefficients were used to initially assess the relationship between CVD risk factors and plasma SPM pathway intermediates and SPM.

Power calculations showed that measurements in at least 20% of participants would be required to give 80% power to detect a correlation coefficient of 0.2 at *p* < 0.05. Consequently, only plasma 18-HEPE, 17-HDHA, 14-HDHA, RvE1 and RvE3 fulfilled these criteria.

Pearson coefficients were calculated separately for each sex for plasma 18-HEPE, 17-HDHA, 14-HDHA, RvE1 and RvE3 and selected omega-3 (EPA, DHA, omega-3 index) and omega-6 (AA) fatty acids, and relevant cardiovascular risk factors (adiposity, alcohol consumption and smoking). Given only RvE1 showed consistent association with cardiovascular risk factors, the effect of BMI (categorized as healthy, overweight, or individuals with obesity), smoking (yes/no) and alcohol consumption (≤100 g/wk v’s >100 g/wk) on plasma RvE1 was assessed using univariate analysis that adjusted for sex differences. This was further explored using multiple regression analysis to assess the significant predictors of plasma RvE1 with a cut-off *p* > 0.1 applied to covariates that were not significantly associated with the outcome. Covariates considered in the model included sex and smoking as categorical variables; BMI, alcohol intake, AA and DHA, (ln) hs-CRP, (ln) triglycerides, cholesterol, adiponectin, and physical activity as continuous variables.

## Results

The characteristics of the participants are shown in Table 1. The study examined 479 men and 499 women of similar age and BMI. Waist circumference, visceral fat volume, systolic and diastolic BP, serum LDL-cholesterol, triglycerides, glucose and liver enzymes were higher in males. The metabolic syndrome was more prevalent in men than women. In contrast, women had higher HDL-cholesterol, hs-CRP, adiponectin and leptin levels, leukocyte and platelet counts, and a larger volume of abdominal subcutaneous fat. Men exercised more, consumed more alcohol and were more likely to be smokers than the women. Women had higher erythrocyte DHA and omega 3 index but lower levels of EPA compared with the men (Table 1). Women using hormonal contraception had higher DHA (4.97% ± 0.07 vs 4.63% ± 0.07, *p* < 0.001) and lower EPA levels (0.84% ± 0.02 vs 0.97% ± 0.02, *p* < 0.001) compared with those not taking hormonal contraception.

**Table 1:** Characteristics of participants studied at the 27-year follow-up of the Raine Study.

Variable		Men (*n* = 479)	Women(*n* = 499)
Age (year)		26.7 ± 0.4	26.7 ± 0.4
BMI (kg/m^2^)		25.9 ± 5.0	25.5 ± 6.2
Waist Circumference (cm)		89.1 ± 13.8	81.0 ± 15.2^a^
Waist/height		0.49 ± 0.07	0.48 ± 0.09
Skinfolds	Subscapular (mm)	17.4 ± 8.6	19.6 ± 9.3^a^
	Abdominal (mm)	24.1 ± 10.7	23.7 ± 9.5
	Suprailiac (mm)	20.3 ± 10.6	21.6 ± 10.0^c^
Systolic BP (mmHg)		119.1 ± 9.2	109.6 ± 9.1^a^
Diastolic BP (mmHg)		65.8 ± 6.4	64.8 ± 6.8^c^
Metabolic Syndrome (%)		9.2	4.3^a^
Total Cholesterol (mmol/L)		4.78 ± 0.89	4.86 ± 0.82
HDL-Cholesterol (mmol/L)		1.30 ± 0.30	1.60 ± 0.38^a^
LDL-Cholesterol (mmol/L)		2.99 ± 0.81	2.82 ± 0.68^a^
*Triglycerides (mmol/L)		0.97 (0.93, 1.01)	0.86 (0.83, 0.90)^a^
Glucose (mmol/L)		4.9 ± 0.6	4.6 ± 0.6^a^
*HOMA-IR		1.17 (1.10, 1.23)	1.18 (1.12, 1.24)
γ glutamyl transpeptidase (U/L)		24.7 ± 20.1	17.6 ± 11.6^a^
Alanine transaminase (U/L)		36.9 ± 24.0	23.8 ± 14.4^a^
Aspartate transaminase (U/L)		30.7 ± 10.4	26.1 ± 9.9^a^
Alkaline phosphatase (U/L)		71.0 ± 19.5	64.2 ± 19.1^a^
*hs-CRP (mg/L)		0.80 (0.73, 1.12)	1.30 (1.16, 1.46)^a^
Leukocyte count (10^9^/L)		6.2 ± 1.8	6.8 ± 1.9^a^
Platelet count (10^9^/L)		249 ± 51	281 ± 63^a^
Adiponectin (µg/ml)		6.3 ± 3.7	10.0 ± 5.0^a^
Leptin (ng/ml)		6.1 ± 10.6	24.7 ± 22.7^a^
Weighted subcutaneous fat volume (cm^3^)		2299 ± 1487	3129 ± 2092^a^
Weighted visceral fat volume (cm^3^)		1446 ± 1001	815 ± 620^a^
Assessment of Lifestyle			
Smoking (%)		23.4%	16.1%^b^
Alcohol intake (g/wk)		160 ± 190	95 ± 148^a^
Physical activity (Mets/wk)		3870 ± 3979	2539 ± 2775^a^
Hormonal contraceptive use (%)		NA	49.7%
Erythrocyte fatty acids			
Arachidonic acid (20:4 n-6) %		17.7 ± 1.4	17.6 ± 1.4
Eicosapentaenoic acid (20:5 n-3) %		1.03 ± 0.48	0.90 ± 0.36^a^
Docosahexaenoic acid (22:6 n-3)%		4.5 ± 1.2	4.8 ± 1.1^a^
Omega 3 Index		5.5 ± 1.5	5.7 ± 1.3^c^

Values are Mean ± SD or *geometric mean and 95% CI. ^a^*p* < 0.001, ^b^*p* < 0.01 ^c^*p* < 0.05 for a sex difference.

Omega 3 index = EPA + DHA content of erythrocytes expressed as a percent of total identified fatty acids.

### Plasma SPM pathway intermediates, SPM and LTB_4_ in males and females

The SPM pathway intermediates (18-HEPE, 17-HDHA and 14-HDHA) were detected in all participants (Table 2). RvE1 and RvE3 were detected in 65% and 55% of participants, respectively. Detectable levels of LTB_4_, LXA_4_ and other SPM in plasma were observed in smaller numbers (4% to 15%) of participants.

**Table 2:** Plasma SPM, SPM pathway intermediates and LTB4 in men and women.

	Men	Women	*P* value for sex difference
AA derived metabolites (pg/ml)			
LTB4	10.4 (6.7,16.0) (*n* = 28)	8.9 (6.0, 13.2) (*n* = 31)	0.60
LXA4	14.5 (11.1, 19.0) (*n* = 38)	9.0 (5.7, 14.2) (*n* = 22)	**0.05**
EPA derived metabolites and E-series resolvins (pg/ml)			
18-HEPE	45.0 (40.6, 49.8) (*n* = 477)	45.1 (44.0, 49.8) (*n* = 498)	0.97
RvE1	6.5 (6.0, 7.1) (*n* = 297)	5.2 (4.9, 5.5) (*n* = 337)	**<0.0001**
RvE2	10.7 (6.5, 16.2) (*n* = 23)	11.4 (8.3, 15.7) (*n* = 23)	0.68
RvE3	19.2 (16.4, 22.3) (*n* = 259)	15.9 (13.8, 18.5) (*n* = 281)	0.09
DHA derived metabolites and D-series resolvins (pg/ml)			
17-HDHA	123.7 (111.9, 136.9) (*n* = 479)	130.1 (117.4, 144.3) (*n* = 499)	**0.01**
RvD1	7.4 (4.2, 13.1) (*n* = 18)	8.7 (5.3, 14.4) (*n* = 27)	0.66
17R-RvD1	9.6 (7.7, 12.1) (*n* = 68)	6.4 (4.5, 9.2) (*n* = 48)	**0.05**
RvD2	6.4 (4.5, 9.0) (*n* = 40)	6.6 (4.0, 11.0) (*n* = 28)	0.91
RvD3	5.2 (4.2,4.6) (*n* = 21)	5.1 (4.1,6.3) (*n* = 21)	0.90
RvD5	2.9 (1.7, 5.1) (*n* = 25)	4.3 (3.0, 6.2) (*n* = 24)	0.25
10S,17S-HDHA	10.4 (7.0, 15.3) (*n* = 70)	12.0 (8.6, 16.7) (*n* = 80)	0.55
PD1	32.5 (20.1, 52.4) (*n* = 52)	48.6 (34.0, 69.3) (*n* = 58)	0.17
DHA derived metabolites and maresins (pg/ml)			
14-HDHA	123.7(111.9, 136.9) (*n* = 479)	130.1 (117.4, 144.3) (*n* = 499)	0.50
MaR1	11.7 (8.8, 15.7) (*n* = 49)	10.5 (8.2, 13.4) (*n* = 51)	0.55

Values are geometric mean and 95% CI.

Bold values indicate significant differences between sexes.

The SPM pathway intermediate 17-HDHA was higher in the women (p = 0.01) whereas the men had higher levels of LXA_4_ (*p* = 0.05), RvE1 (*p* < 0.0001) and 17R-RvD1 (*p* = 0.05) (Table 2). Sex differences were not significant for other SPM pathway intermediates (18-HEPE and 14-HDHA) or other E- and D- series resolvins and maresin-1.

#### Effect of hormonal contraception

Women taking hormonal contraceptives had lower levels of 18-HEPE (39.9 pg/ml, CI 34.2, 46.6) compared with those not taking hormonal contraceptives (51.1 pg/ml, CI 44.7, 57.7; *p* = 0.016). Hormonal contraceptive use in women did not significantly affect levels of 17-HDHA or 14-HDHA or any other SPM (*p* > 0.1, data not shown).

### Relationships between plasma SPM and erythrocyte fatty acids and CVD risk factors

Analyses examining the relationship between the SPM and CVD risk factors from Table 1 were confined to the SPM pathway intermediates, RvE1 and RvE3. Correlation coefficients were calculated separately for each sex (Table 3).

**Table 3:** Pearson correlation coefficients ® for SPM pathway intermediates, RvE1 and RvE3.

	18-HEPE	RvE1	RvE3	17-HDHA	14-HDHA
Men	*n* = 479	*n* = 297	*n* = 259	*n* = 479	*n* = 479
Women	*n* = 499	*n* = 337	*n* = 281	*n* = 499	*n* = 499
Erythrocyte Arachidonic acid (20:4 n6) %					
Men	−0.13, *p* = 0.001	−0.16, *p* = 0.001	NS	−0.13, *p* = 0.001	−0.15, *p* = 0.001
Women	−0.12, *p* = 0.008	−0.22, *p* < 0.0001	NS	−0.10, *p* = 0.023	−0.13, *p* < 0.0001
Erythrocyte Eicosapentaenoic acid (20:5 n3) %					
Men	0.31, *p* < 0.0001	NS	0.15, *p* = 0.014	0.22, *p* < 0.0001	0.21, *p* < 0.0001
Women	0.22, *p* < 0.0001	NS	0.06 NS	0.09, *p* = 0.05	0.10, *p* = 0.03
Erythrocyte Docosahexaenoic acid (22:6 n3) %					
Men	NS	−0.10, *p* = 0.07	NS	0.21, *p* < 0.0001	0.21, *p* < 0.0001
Women	NS	−0.10 *p* = 0.06	NS	0.11, *p* = 0.012	0.13, *p* = 0.004
Omega 3 index %					
Men	NS	−0.10, *p* = 0.07	NS	0.24, *p* < 0.0001	0.23, *p* < 0.0001
Women	NS	−0.10 *p* = 0.06	NS	0.12, *p* = 0.007	0.14, *p* = 0.002
Alcohol intake (g/wk)					
Men	NS	0.28, *p* < 0.0001	NS	NS	NS
Women	NS	0.23, *p* = 0.001	NS	NS	NS
BMI (Kg/m^2^)					
Men	NS	−0.12, *p* = 0.03	−0.03 NS	NS	NS
Women	NS	−0.08 NS	−0.12, *p* = 0.047	NS	NS
Waist Circumference (cm)					
Men	NS	−0.10, *p* = 0.07	NS	−0.07 NS	−0.07 NS
Women	NS	−0.12, *p* = 0.03	NS	−0.13, *p* = 0.003	−0.10, *p* = 0.023
Waist/Height (m/m)					
Men	NS	−0.10, *p* = 0.07	NS	−0.06 NS	−0.06 NS
Women	NS	−0.12, *p* = 0.03	NS	−0.14, *p* = 0.002	−0.10, *p* = 0.023
Weighted abdominal subcutaneous fat volume (cm^3^)					
Men	NS	−0.12, *p* = 0.05	NS	NS	NS
Women	NS	−0.06 NS	NS	NS	NS
SKINFOLD THICKNESS:		subscapular (mm)			
Men	NS	−0.12, *p* = 0.04	NS	NS	NS
Women	NS	−0.09 NS	NS	NS	NS
abdominal (mm)					
Men	NS	−0.12, *p* = 0.04	NS	NS	NS
Women	NS	−0.07 NS	NS	NS	NS
suprailiac (mm)					
Men	NS	−0.16, *p* = 0.007	NS	NS	NS
Women	NS	−0.08 NS	NS	NS	NS

SPM and SPM pathway intermediates and alcohol intake were log transformed for analysis.

#### SPM pathway intermediates

There were significant relationships between SPM pathway intermediates and erythrocyte fatty acids. In both men and women 18-HEPE, 17-HDHA and 14-HDHA were positively correlated with EPA (*p* < 0.05) and negatively correlated with AA (*p* < 0.05), whereas DHA was positively correlated with 17-HDHA and 14-HDHA (*p* < 0.02) but not 18-HEPE (Table 3). Omega 3 index was positively correlated with 17-HDHA and 14-HDHA in both sexes (*p* < 0.0001). In women but not men, 17-HDHA and 14-HDHA were negatively correlated with waist circumference and waist/height ratio (*p* < 0.05) (Table 3). There were no significant correlations in either sex between 18-HEPE, 17-HDHA and 14-HDHA and any of the other risk factor variables shown in Table 1.

#### RvE1 and RvE3

In both men and women RvE1 was negatively correlated with AA (*p* < 0.01) and DHA (*p* = 0.07) Table 3. RvE1 was negatively correlated with a range of measures of adiposity in men and women including waist circumference (*r* = −0.10, *p* = 0.07; and *r* = −0.12, *p* = 0.03, respectively), waist/height ratio (*r* = −0.10 *p* = 0.07 and *r* = -0.12 *p* = 0.03, respectively), (BMI *r* = −0.12, *p* = 0.05 in women) and abdominal subcutaneous fat (*r* = −0.12 *p* = 0.05 in men). There was a negative relationship between RvE1 and skinfold thickness (abdominal, subscapular and suprailiac) that was most pronounced in men (Table 3). RvE1 was positively correlated with alcohol intake in both sexes (*p* < 0.01) (Table 3). RvE1 was not significantly correlated with systolic BP (males: *r* = 0.036, *p* = 0.53; and females *r* = 0.068, *p* = 0.212).

RvE3 was positively correlated with EPA (*r* = 0.15, *p* < 0.05) in men and negatively correlated with BMI (*r* = −0.12, *p* < 0.05) in women (Table 3).

There were no significant correlations between RvE1 or RvE3 with measures of inflammation (hs-CRP and leukocyte count), or any of the other risk factor variables shown in Table 1.

### Effect of obesity, smoking and alcohol consumption on plasma RvE1

**Fig. 1:**
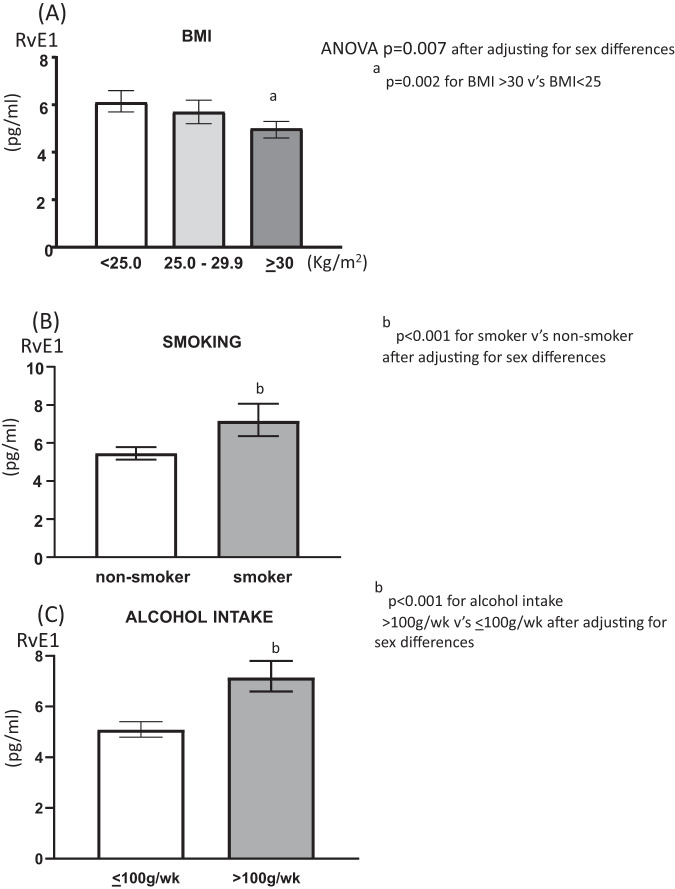
The effect of obesity smoking and alcohol consumption on plasma RvE1. Means and 95% CI for RvE1 according to differences in (**A**) BMI, (**B**) smoking and (**C**) alcohol consumption.

The effect of obesity on RvE1 was further examined by classifying BMI into three categories: healthy individuals (<25 kg/m^2^), overweight individuals (25–29.9 kg/m^2^) or individuals with obesity (≥30 kg/m^2^). After adjusting for sex differences, there was a significant effect of BMI on plasma RvE1 levels (*p* = 0.007) such that individuals with obesity had lower levels of RvE1 compared with those in the healthy weight range (*p* = 0.002) (Fig. 1A). Smokers were found to have significantly higher levels of RvE1 than non-smokers (*p* < 0.001) (Fig. 1B). Alcohol intake was categorized based on the Australian standards that recommend restricting alcohol intake to a maximum of 10 standard drinks a week (100 g alcohol/wk) in order to reduce the lifetime risk of harm from alcohol-related disease or injury [23]. Alcohol intake above 100 g/wk associated with significantly increased RvE1 (*p* = 0.001) (Fig. 1C). The effect of obesity (*p* < 0.006), smoking (*p* < 0.001) and alcohol consumption (*p* < 0.001) on RvE1 remained significant after further adjusting for the inflammatory markers hs-CRP and leukocyte count. Examining the effects of obesity, smoking and alcohol on RvE1 in men and women separately showed a similar trend to the group as a whole.

In multiple regression analysis, the significant predictors of RvE1 were female sex (*β* = −0.144, *p* = 0.006); BMI (*β* = −0.104, *p* = 0.044); AA (*β* = −0.171, *p* = 0.001); DHA (*β* = −0.111, *p* = 0.037); alcohol intake (β = 0.177, *p* = 0.001) and smoking (*β* = 0.109, *p* = 0.042). The model accounted for 14% of the variance in plasma RvE1 (Table 4). Substituting different measures of adiposity (waist circumference, *β* = −0.111, *p* = 0.046; waist/height, *β* = −0.099, *p* = 0.055 or abdominal subcutaneous fat *β* = −0.096, *p* = 0.08) gave similar results but the model that included BMI explained more of the variance in RvE1. Visceral fat was not a significant predictor in the model (*β* = −0.83, *p* = 0.175).

**Table 4:** Regression model examining predictors of plasma RvE1.

Dependent variable	Predictor variables	*β*	*t*	*p*-value
(ln) Plasma RvE1 (pg/ml)				
	Sex (female)	−0.144	−2.740	0.006
	BMI	−0.104	−2.023	0.044
	Smoker	0.109	2.043	0.042
	(ln) Alcohol intake	0.177	3.218	0.001
	Arachidonic acid	−0.171	−3.334	0.001
	Docosahexaenoic acid	−0.111	−2.093	0.037

Adjusted *r*^2^ = 0.14, ANOVA_F6,520_ = 14.00, *p* < 0.001.

Sex and smoking were entered as categorical, BMI (kg/m^2^), alcohol intake (g/wk), arachidonic acid (%) and docosahexaenoic acid (%) were entered as continuous variables. (ln) represents the natural logarithm transformation. Other predictors variables entered in the backward regression model were (ln) HsCRP (mg/L), (ln) triglycerides (mmol/L), cholesterol (mmol/L), (ln)adiponectin µg/ml, and physical activity (mets/wk).

## Discussion

This is the first large population study to examine plasma SPM in relation to sex differences, fatty acid status and selected relevant lifestyle and cardiovascular risk factors. The main finding from this study relates to the lifestyle risk factors and their relationship with plasma RvE1 levels. We found that plasma RvE1 was consistently negatively associated with a range of measures of adiposity including BMI, waist circumference, waist-to-height ratio, abdominal subcutaneous fat volume and skinfold thickness (subscapular, abdominal and suprailiac) in both men and women. In particular, plasma RvE1 was significantly lower in individuals with obesity (BMI ≥ 30 kg/m^2^) compared with those in the healthy weight range. Our results from this study add further support for a role for RvE1 in inflammation resolution in overweight individuals and are supported by observations that there are a number of SPM including RvE1 present in human adipose tissue [26], with deficiencies in SPM described in both animals and humans with obesity [27–30]. In addition, we previously showed that modest weight loss in overweight humans resulted in an increase neutrophil RvE1 [17]. Together these finding suggest that RvE1 may be particularly sensitive to changes in body fat [17].

RvE1 was first identified in vivo during the resolution phase of inflammation in exudates from murine dorsal pouches treated with aspirin and EPA [31] Studies also showed the interaction of human leukocytes with endothelial cells within the vasculature rapidly converted 18-HEPE to RvE1 via transcellular biosynthesis [31]. The latter actions of RvE1 may be relevant to the present findings in view of the critical role of vascular endothelial cell interactions with human leukocytes in atherosclerosis and CVD risk. Other reports have confirmed RvE1 is present in human plasma, biological fluids and tissues, and a number of human diseases and conditions [32].

The finding that plasma RvE1 was elevated in smokers has not been previously reported and may be a homeostatic response to counter inflammation that is known to occur in smokers [33]. There is some support for this argument from the work by Takamiya et al. who showed that RvE1 maintained macrophage function under cigarette smoke induced oxidative stress [34].

Increasing alcohol consumption also associated with increased plasma RvE1. Alcohol has the potential to alter synthesis of SPMs by affecting the availability of fatty acid substrates [35] and the activity of COX-2 [36] and 5-lipoxygenase [37], both key enzymes involved in SPM synthesis. In a randomized controlled trial, we previously showed that men drinking red wine (41 g/alcohol/day) for 4 weeks had elevated plasma levels of 18-HEPE, RvD1 and 17-R-RvD1 [38]. However, in a controlled trial of red wine in Type 2 diabetes, we found no difference between plasma levels of SPM after red wine consumption for 4 weeks [39]. The lack of effect of alcohol on SPM in that study was postulated to be due to the lower alcohol intake and the diabetic patient population whose baseline levels of SPM were elevated when compared with healthy controls [39].

The significant negative association of RvE1 with both AA and DHA is interesting. These fatty acids are substrates for other pathways involved in inflammation resolution (LXA4 and D-series resolvins, respectively) and may play a part in regulating RvE1 levels during inflammation resolution. The SPM pathway intermediates were also positively correlated with their precursor fatty acids EPA and DHA and negatively correlated with AA, suggesting a degree of complexity in the regulation of SPM pathway intermediates. In contrast, the RvE3 and the D-series resolvins were not significantly related to EPA and DHA, respectively.

We detected SPM pathway intermediates (18-HEPE, 17-HDHA, and 14-HDHA) in plasma of all the young adults. SPM, such as RvE1 and RvE3, were detected in 65% and 55% of participants, respectively. LTB4, LXA4 and other SPM were detected in 4–15% of participants. Our findings hold relevance in view of the large sample size (approximately 1000 individuals) and consistent with observations in smaller studies that showed that not all SPM are present in human plasma [40–44], likely reflecting the autacoid nature of SPM and/or differences in the patient populations studied. Differences between levels in men and women were apparent for 17-HDHA, and three SPM (RvE1, 17R-RvD1 and LXA_4_). Hormonal contraceptive use in women resulted in reduced levels of 18-HEPE which may in part be related to their reduced levels of the fatty acid precursor EPA. Hormonal contraception did not significantly affect other SPM pathway intermediates or SPM.

A strength of the study is that it is a large phenotypically well defined population cohort, studied within a narrow age range. We acknowledge that the cohort was predominantly Caucasian; there was attrition in the cohort from birth to the 27-year survey that we studied. However, the overall demographic characteristics of individuals studied have not been found to be significantly different from the general population in Western Australia [45].

In conclusion, we have shown that plasma RvE1 is affected by sex differences and lifestyle factors that are relevant to the risk of cardiovascular and other chronic diseases. The lower levels of RvE1 in individuals with obesity may be integral to the underlying inflammation that closely associates with obesity. The association between RvE1 and alcohol consumption and smoking, requires further study but in healthy young adults could reflect a homeostatic response to counter inflammation associated with these risk factors.

### Supplementary information

Supplementafigure 1–6

## Data Availability

The datasets generated during and/or analyzed during the current study are not available. The Raine study is committed to a high level of confidentiality of the data in line with the informed consent provided by participants. Requests for data should be directed to the Raine Study Executive.
