# Supplementary material for: Adiposity associates with lower plasma resolvin E1 (Rve1): a population study
Source: Int J Obes (Lond). 2024 Feb 12;48(5):725–32. doi: 10.1038/s41366-024-01482-x (PMC11058310; doi:10.1038/s41366-024-01482-x)
Supplement: Supplementary file 1 — Supplementafigure 1–6 [file 41366_2024_1482_MOESM1_ESM.pdf]

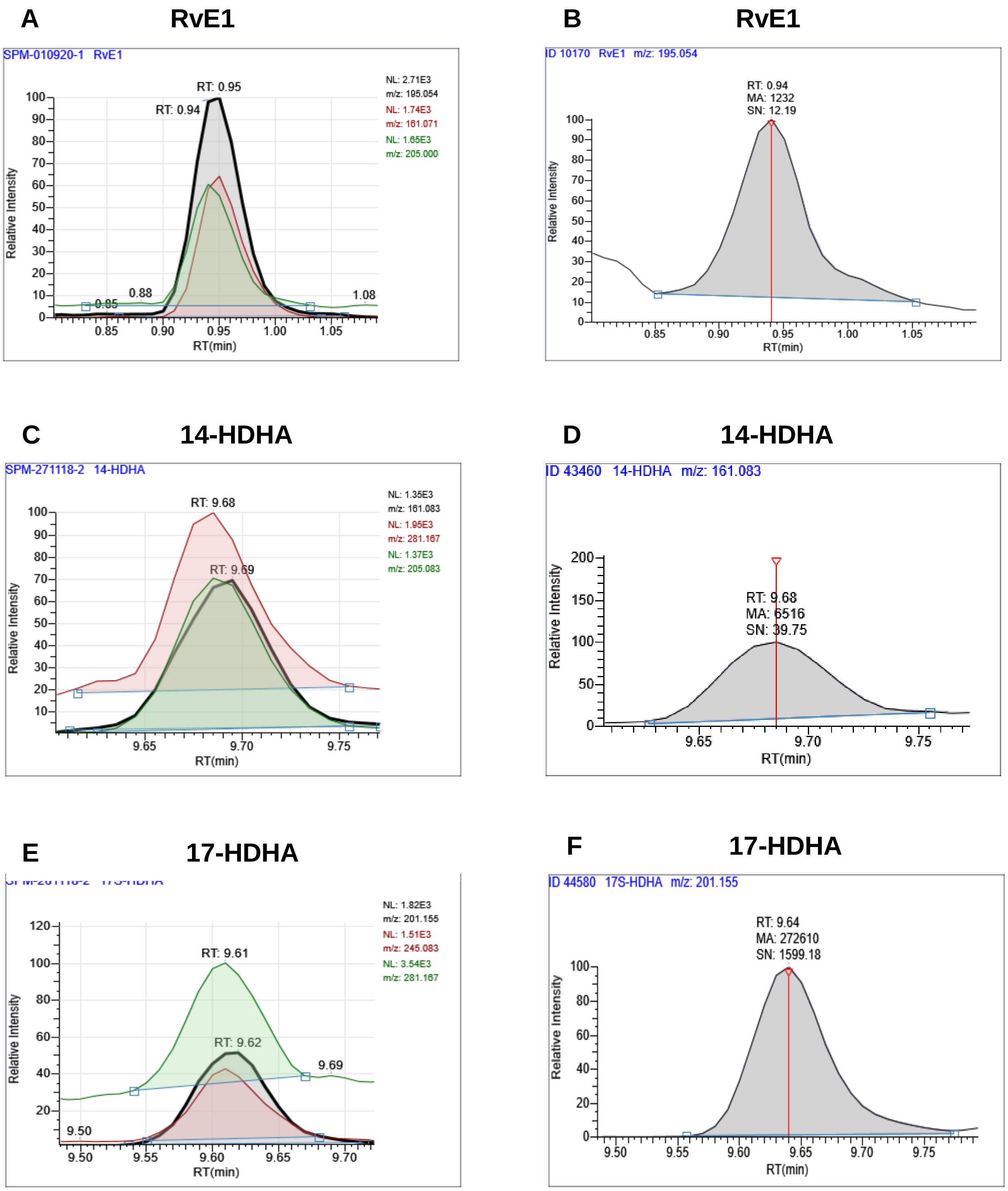

Supplementary Figure 1. Representative SRM chromatograms of resolvin E1 (RvE1), 14-hydroxydocosahexaenoic acid (14-HDHA) and 17-hydroxydocosahexaenoic acid (17-HDHA) by liquid chromatography-tandem mass spectrometry (LCMSMS) and monitored in negative ion mode: (A) standard of RvE1 with ion overlay of transitions *m/z* 349.125 → 195.054 (*quantifying ion*, *black*), *m/z* 161.071 (qualifying ion, *red*) and *m/z* 205.000 (qualifying ion, *green*); (B) RvE1 measured in a representative plasma; (C) standard of 14-HDHA with overlay of transitions *m/z* 343.175 → 161.083 (*quantifying ion*, *black*), *m/z* 281.167 (qualifying ion, *red*) and *m/z* 205.083 (qualifying ion, *green*); (D) 14-HDHA measured in a representative plasma; (E) standard of 17-HDHA with ion overlay of transitions *m/z* 343.188 → 201.155 (*quantifying ion*, *black*), *m/z* 245.083 (qualifying ion, *red*) and *m/z* 281.167 (qualifying ion, *green*); (F) 17-HDHA measured in a representative plasma.

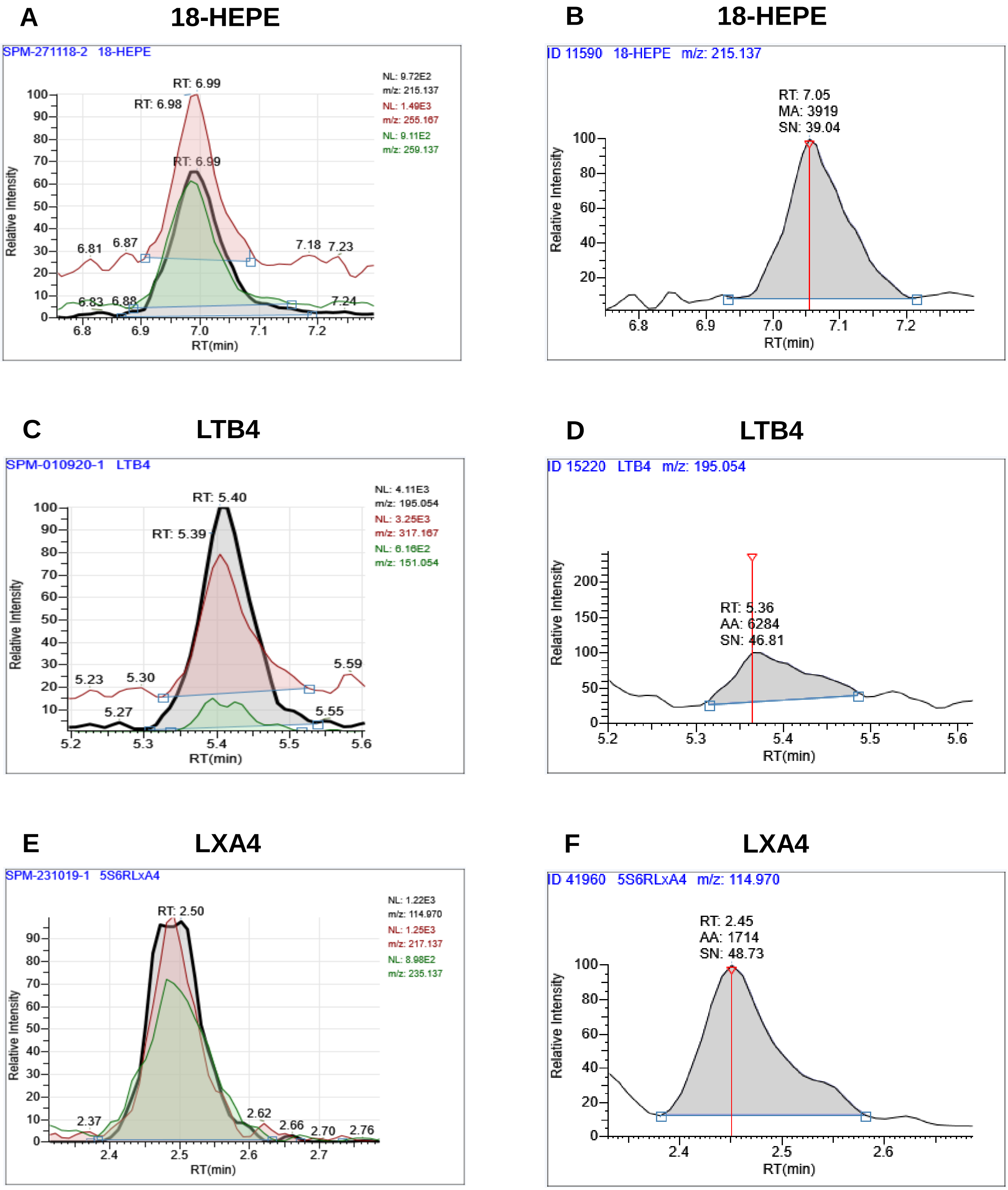

Supplementary Figure 2. Representative SRM chromatograms of 18-hydroxyeicosapentaenoic acid (18-HEPE), leukotriene B4 (LTB4) and lipoxin A4 (LXA4) by liquid chromatography-tandem mass spectrometry (LCMSMS) and monitored in negative ion mode: (A) standard of 18-HEPE with ion overlay of transitions *m/z* 317.200→215.137 (*quantifying ion, black*), *m/z* 255.167 (qualifying ion, *red*) and *m/z* 259.137, (qualifying ion, *green*); (B) 18-HEPE measured in a representative plasma; (C) standard of LTB4 with overlay of transitions *m/z* 335.175→ 195.054 (*quantifying ion, black*), *m/z* 317.167 (qualifying ion, *red*) and *m/z* 151.054 (qualifying ion, *green*); (D) LTB4 measured in a representative plasma; (E) standard of LXA4 with ion overlay of transitions *m/z* 351.188→114.970 (*quantifying ion, black*), *m/z* 217.137 (qualifying ion, *red*) and *m/z* 235.137 (qualifying ion, *green*); (F) LXA4 measured in a representative plasma.

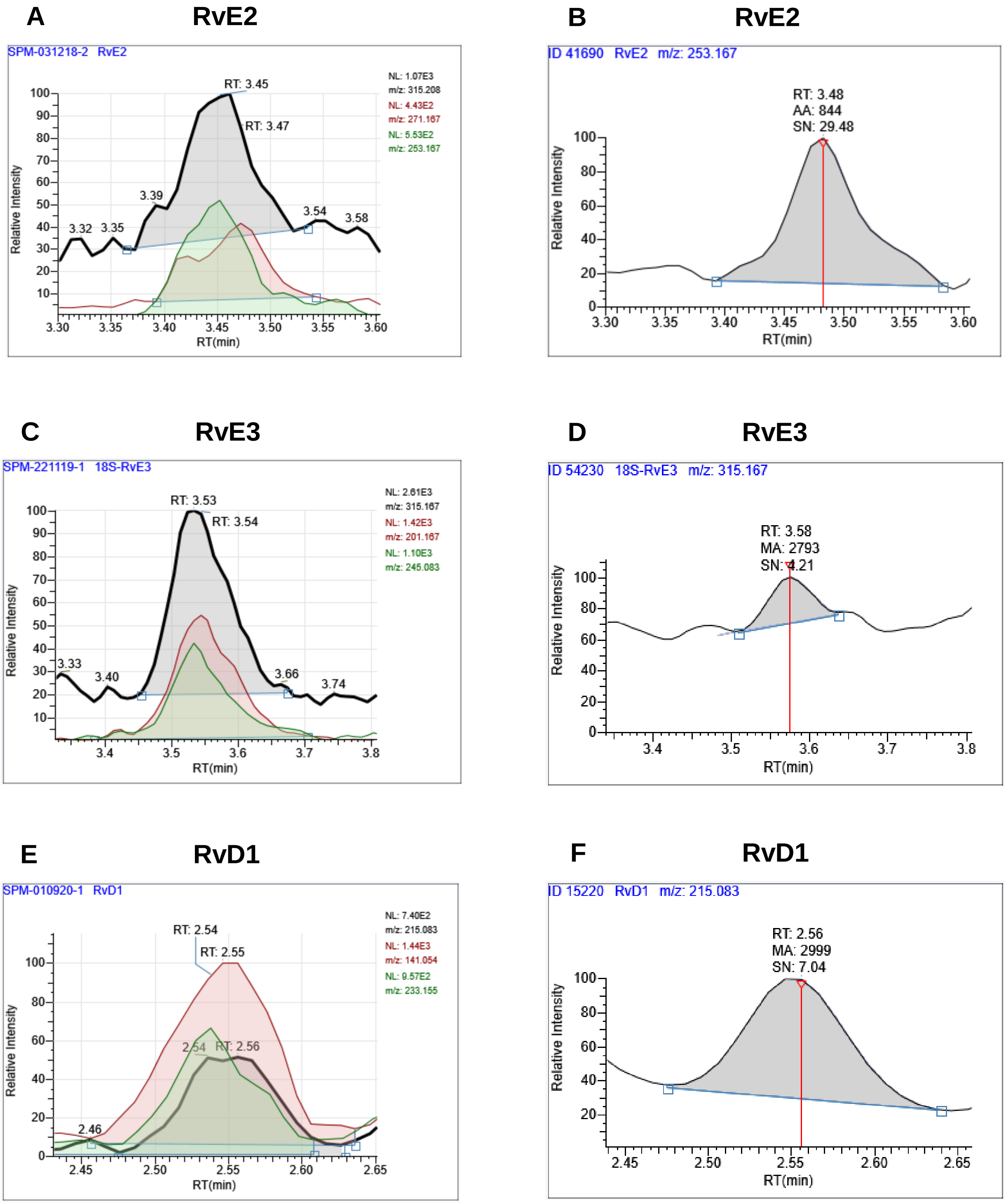

Supplementary Figure 3. Representative SRM chromatograms of resolvin E2 (RvE2), resolvin E3 (RvE3) and resolvin D1 (RvD1) by liquid chromatography-tandem mass spectrometry (LCMSMS) and monitored in negative ion mode: (A) standard of RvE2 with ion overlay of transitions *m/z* 333.175→ 253.167 (*quantifying ion*, *black*), *m/z* 315.208 (qualifying ion, *red*) and *m/z* 271.167 (qualifying ion, *green*); (B) RvE2 measured in a representative plasma; (C) standard of RvE3 with overlay of transitions *m/z* 333.225→315.167 (*quantifying ion*, *black*), *m/z* 201.167 (qualifying ion, *red*) and *m/z* 245.083 (qualifying ion, *green*); (D) RvE3 measured in a representative plasma; (E) standard of RvD1 with ion overlay of transitions *m/z* 375.200→ 215.083 (*quantifying ion*, *black*), *m/z* 141.054 (qualifying ion, *red*) and *m/z* 233.155 (qualifying ion, *green*); (F) RvD1 measured in a representative plasma.

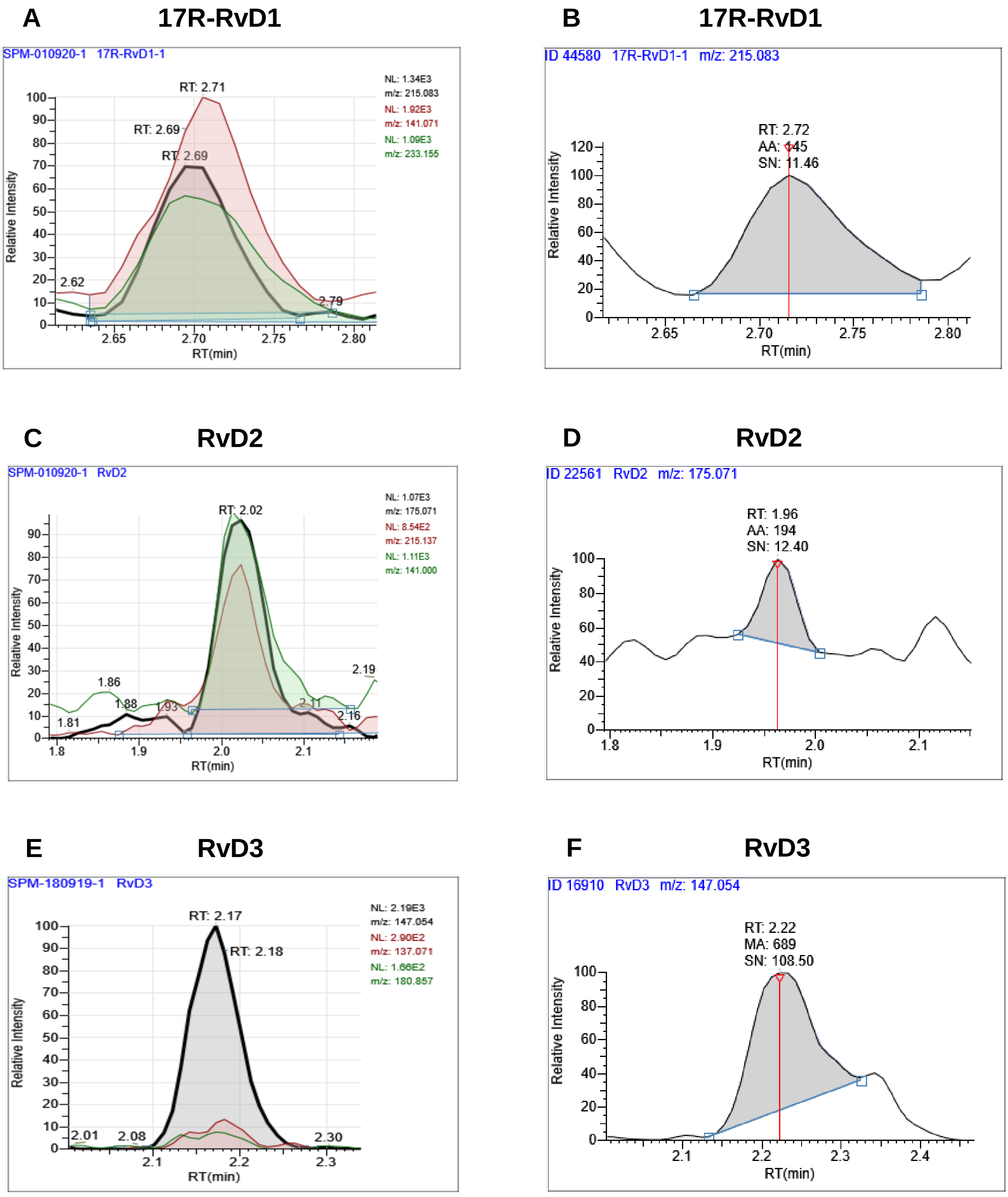

Supplementary Figure 4. Representative SRM chromatograms of 17R-resolvin D1 (17R-RvD1), resolvin D2 (RvD2) and resolvin D3 (RvD3) by liquid chromatography-tandem mass spectrometry (LCMSMS) and monitored in negative ion mode: (A) standard of 17R-RvD1 with ion overlay of transitions *m/z* 375.150→215.083 (*quantifying ion*, *black*), *m/z* 141.071 (qualifying ion, *red*) and *m/z* 233.155 (qualifying ion, *green*); (B) 17R-RvD1 measured in a representative plasma; (C) standard of RvD2 with overlay of transitions *m/z* 375.200→175.071 (*quantifying ion*, *black*), *m/z* 215.137 (qualifying ion, *red*) and *m/z* 141.000 (qualifying ion, *green*); (D) RvD2 measured in a representative plasma; (E) standard of RvD3 with ion overlay of transitions *m/z* 375.138→147.054 (*quantifying ion*, *black*), 137.071 (qualifying ion, *red*) and 180.857 (qualifying ion, *green*); (F) RvD3 measured in a representative plasma.

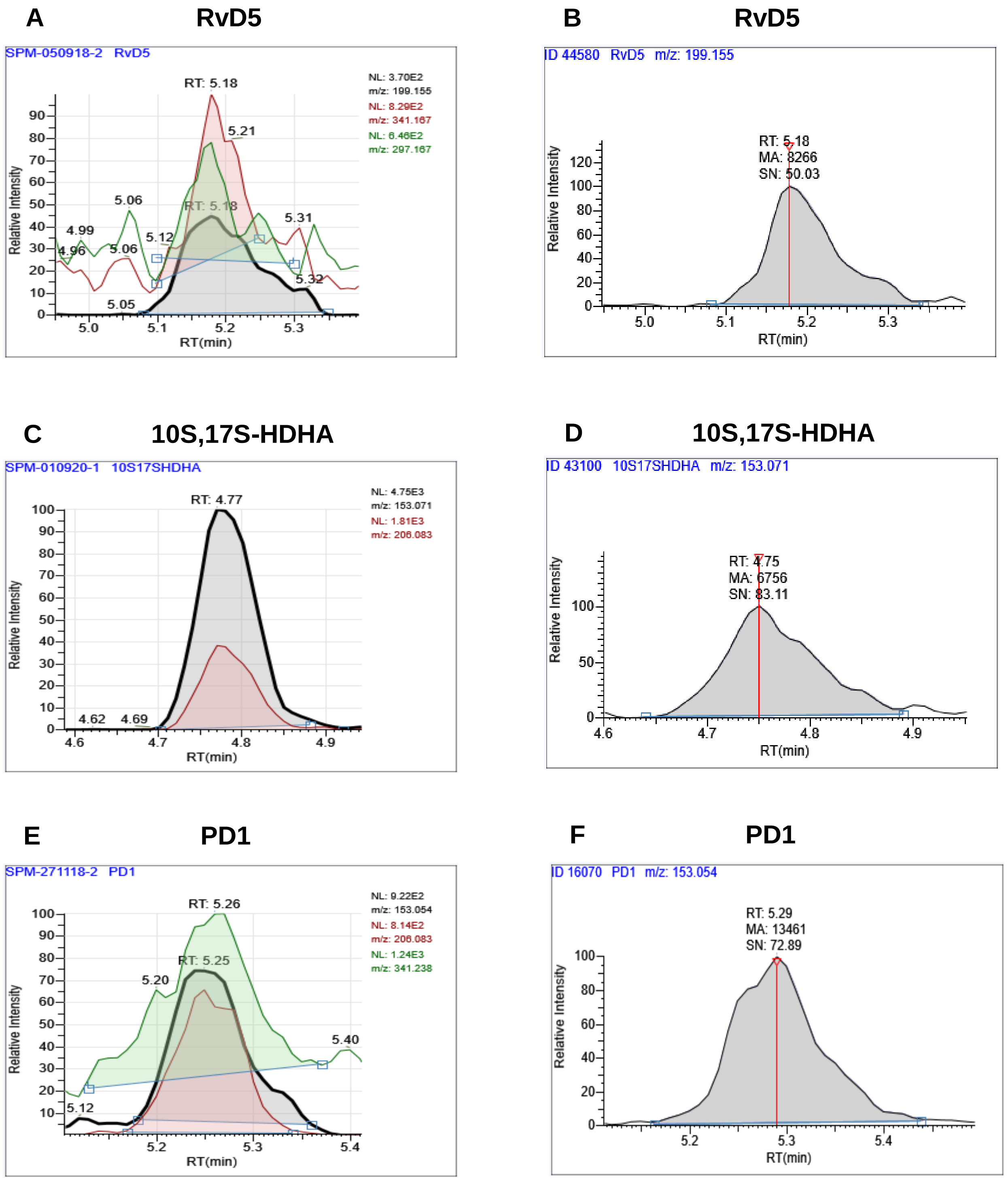

Supplementary Figure 5. Representative SRM chromatograms of resolving D5 (RvD5), 10S,17S-dihydroxy-docosahexaenoic acid (10S,17S-HDHA) and protectin D1 (PD1) by liquid chromatography-tandem mass spectrometry (LCMSMS) and monitored in negative ion mode: (A) standard of RvD5 with ion overlay of transitions *m/z* 359.125→199.155 (*quantifying ion*, *black*), *m/z* 341.167 (qualifying ion, *red*) and *m/z* 297.167 (qualifying ion, *green*); (B) RvD5 measured in a representative plasma; (C) standard of 10S,17S-HDHA with overlay of transitions *m/z* 359.212→153.071 (*quantifying ion*, *black*), *m/z* 206.083 (qualifying ion, *red*); (D) 10S,17S-HDHA measured in a representative plasma; (E) standard of PD1 with ion overlay of transitions *m/z* 359.162→153.054 (*quantifying ion*, *black*), 206.083 (qualifying ion, *red*) and 341.238 (qualifying ion, *green*); (F) PD1 measured in a representative plasma.

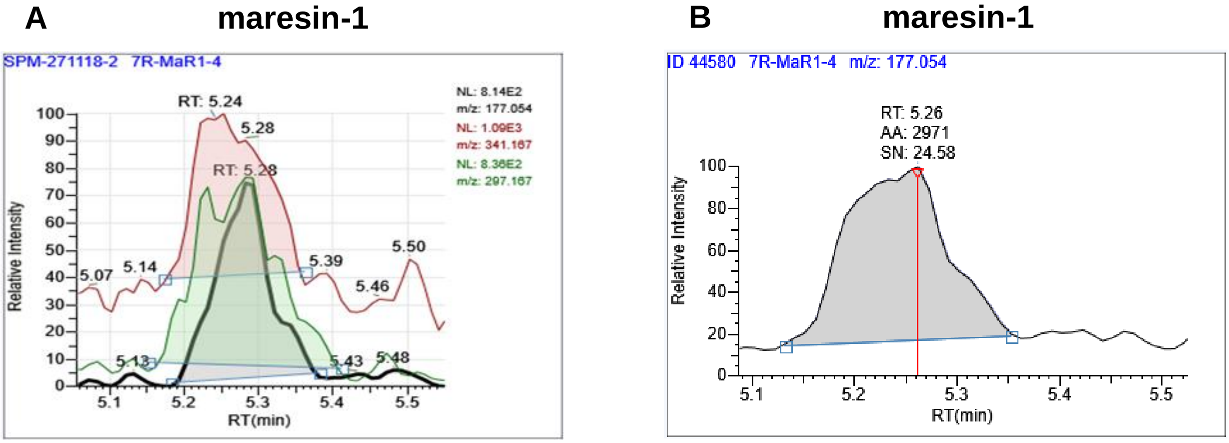

Supplementary Figure 6. Representative SRM chromatograms of maresin-1 (MaR1) by liquid chromatography-tandem mass spectrometry (LCMSMS) and monitored in negative ion mode: (A) standard of MaR1 with ion overlay of transitions *m/z* 359.212→177.054 (*quantifying ion*, *black*), 341.167 (qualifying ion, *red*) and 297.167 (qualifying ion, *green*) (B) MaR1 measured in a representative plasma.
